# Advanced laser pulse metrology through 2D self-referenced spectral interferometry

**DOI:** 10.1038/s41598-025-02813-2

**Published:** 2025-05-22

**Authors:** Thomas Oksenhendler, Stefan Bock, Jörn Dreyer, René Gebhardt, Uwe Helbig, Toma Toncian, Ulrich Schramm

**Affiliations:** 1 iTEOX, Gometz-le-châtel, 91940 France; 2 Helmoltz-Zentrum Dresden-Rossendorf (HZDR), Bautzner Landstr. 400, 01328 Dresden, Germany; 3 Technische Universität Dresden, 01062 Dresden, Germany

**Keywords:** Ultrafast photonics, Optical metrology, Optics and photonics, Ultrafast lasers

## Abstract

The temporal contrast requirements of high-power laser pulses are growing with the ongoing increase of available intensities. Novel measurement concepts in the far-field domain capable of providing information on the origin of pre-pulses or coherent and incoherent pedestals are thus needed with single-shot and full-spectral band measurement capabilities. Advancements of the 2D self-referenced spectral interferometry (SRSI) method represent one solution to this need. Here, we discuss experimental progress together with the theoretical potential and limitations of this approach. The dynamic characteristics of measurements in single-shot-mode and through accumulation of shots is used to measure coherent and incoherent temporal contrast features on dynamic levels of up to 90 dB and a temporal range of up to 100 ps and with 25 fs resolution. This unprecedented performance is validated through the measurement of coherent pre-pulses below the incoherent amplified spontaneous emission (ASE) level and the determination of their spectrograms.

## Introduction

When interacting with solid state matter, ultra-intense ultra-short laser pulses enable a wide range of fundamental and applied research in the fields of high energy density science and inertial confinement fusion^1–4^ as well as relativistic laser plasma and advanced accelerator physics^5–9^. To exploit the extreme peak power of such laser pulses to their full extent, great care has to be invested into the control of the temporal intensity contrast, the metric for the temporal rise of the laser pulse with respect to its peak intensity. Premature ionization of any solid-state target and subsequent pre-plasma formation and expansion strongly affects the outcome of the interaction with the main pulse^4,7,10,11^ and thus has to be controlled - in particular for the case of plasma accelerator development - based on knowledge of the on-shot pulse properties^12,13^. Latest high intensity short-pulse laser projects reach the 10 PW peak power range and intensities>10$^{23}$ W/cm² in focus^14^ and thus require contrast ratios in the range of 10$^{-12}$ or better on temporal scales between ns and few 10 ps to few ps prior to the main pulse and corresponding on-shot metrology. These exceptional requirements have driven the development of new metrology concepts in parallel to substantial efforts in temporal contrast improvement^15–21^ with a list of metrology boundary conditions presented in the following. An application critical constraint results from the low repetition rate of most large laser systems (<1 Hz) and shot-to-shot fluctuations of key beam parameters. Thus, single-shot metrology capability is preferred^22,23^. Higher order pulse distortions like spatial-temporal couplings differently impact metrology in near field and far field and have to be accounted for^24^. As the key parameter on target is the focal intensity, measurements are ideally performed in the far field or at least should yield pulse contrast information in the far field. Further, the broad spectral band-width of short pulse lasers needs to be fully covered or step-wise resolved by the measurement technique^25^. For high dynamic range contrast measurement diagnostics commonly used rely on cross-correlators. These single-shot or scanning third- or fourth-order correlators exploit intensity driven effects and cannot recover amplitude and phase of the signal in the temporal domain. Moreover, the measurement is often performed in an unspecified midfield region and compared to experimental conditions using the focused (far field) beam, inevitably loosing information^24^. In addition, due to harmonic generation in nonlinear crystals, the temporal resolution of these instruments is low compared to the original pulse duration. Spectral information can be lost in the process, misrepresenting the full pulse characteristics^25^. To improve pulse contrast of large laser systems based on its measurement, additional information apart from mere intensity contrast level is required for pre-pulses and pedestals, such as coherence, spectral content, or spatial-temporal properties. The apparent need for a measurement range of the order of 100 ps and covering at least a dynamic range, defined by the ratio between the maximum and the minimum measurable signal strength (not to be confused with the background noise level), of 10$^8$ (80 dB), experienced in laser-solid interaction experiments, led to the introduction of an extended SRSI concept in 2017^26^ which is summarized in the following and represents the basis for its further development reported here. Compared to the original setup having achieved an intensity dynamic of 85 dB in a temporal window of 18 ps, here, this window could be widened to 120 ps featuring a slightly enhanced dynamic of 90 dB at the center of the window. Self-referenced spectral interferometry with extended time excursion (SRSI-ETE, hereafter referred to as “2D SRSI”) is based on the recording of the two-dimensional interferogram of tilted beams instead of the previous co-linear geometry. It thereby avoids ambiguities in the reconstruction of the temporal signature, limiting the temporal range by spectral resolution only, and increasing dynamic range due to the increased information multiplicity in the two-dimensional detector. It extended the time window to 16 ps with a dynamic range of up to 80 dB on a single pulse basis. In this article we introduce a second-generation setup capable of characterizing the coherent contrast pedestal on single shot basis over a window of 120 ps and exceeding the 80 dB sensitivity in maximum. With the availability of such a device, single shot contrast characterization within the range critical for experiments and correlation with the experimental outcome is in reach.

## 2D self-referenced spectral interferometry

**Fig. 1:**
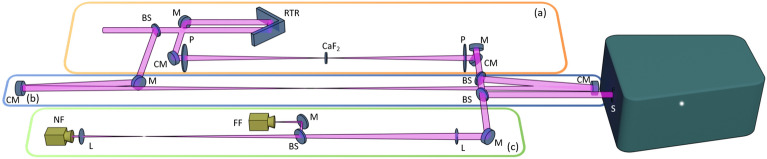
2D SRSI setup. (**a**) the incident beam is split at a beamsplitter (BS). The main part is transmitted and can be delayed by a movable retro-reflector (RTR) and used for XPW reference generation (CaF$_2$) between crossed polarizers (P) and focusing by curved mirrors (CM). (**b**) the reflected beam is imaged by CM from the entrance onto the spectrometers entrance slit (S). (**c**) for alignment purpose a farfield (FF) and nearfield (NF) camera are integrated.

For the final optimization of pulse compression at high peak power ultra-short pulse laser systems, the method of self-referenced spectral interferometry (SRSI)^27^ can commonly be applied. This principle finds application in the broadly used commercially available “Wizzler”^28,29^ by Fastlite. Its on-shot responsivity and its high dynamic range of up to 50 dB opened state-of-the-art real-time higher order spectral phase correction capability^30^ in combination with acousto-optical programmable dispersive filters^31^ (typically Fastlite’s Dazzler) and thus improves pulse compression quality and contrast up to its temporal measurement range of few ps. In detail, the ability of this method to accurately determine the spectral phase, even on the edges of the spectrum and with higher sensitivity and dynamic range than the spectrometer itself, is specific to its homodyne detection principle^28,32^. SRSI in its commercial form (Wizzler) uses a compact 1D spectrometer. Improving the temporal window and dynamic range of this concept, SRSI was extended to 2D exploiting a spatially imaging spectrometer^26^. In this paper we present a novel implementation of the latter extension with a greatly enhanced temporal range of up to 120 ps while maintaining the temporal resolution, and a boosted dynamic range of up to 90 dB. Before exploring the high dynamic range capabilities of the revisited SRSI scheme, it is helpful to recall the 2D SRSI method (for details, see^26^). The pulse to be measured is separated in two in an interferometer setup (fig.1). One of the replicas propagates to the entrance of the spectrometer while remaining essentially identical to the initial pulse (e.g. by imaging the entrance of the interferometer onto the spectrometer’s entrance). The other arm of this interferometer contains a temporal and spatial filtering device allowing the self-generation of a reference. Spectral interferometry^33^ allows a direct determination of the spectral phase difference between the pulse to be measured and the reference by a signal processing implementation using fast Fourier transforms^34^. The key point is therefore to have a reference featuring a broader spectrum as the initial pulse with known (ideally flat) spectral phase and a smooth spatial distribution. Achieving this goal requires a spatial and temporal nonlinear filter, realized e.g. through applying the XPW (nonlinear cross-polarized wave) effect to a focused beam. Since this filter acts smoothingly simultaneously on the temporal and spatial aspects, the output suits as a spatial, temporal, spectral and spatial-temporal reference. The generated reference and the signal pulses are superimposed on the entrance slit of the 2D spectrometer, imaging the entrance slit spectrally resolved onto the detector plane, with an angle $\alpha$ and time delay $\tau$ in between both pulses. The resulting interferogram is in consequence tilted and shows modulations in the spectral and spatial domain. The 2D Fourier transform of this tilted spatial-spectral interferogram gives non-overlapped access to the DC and AC terms, separated in the Fourier domain, or k-t-space, due to the 2D characteristics. The standard SRSI algorithm^32^ can be used to recover the temporal amplitude and phase or directly obtain the temporal contrast, thanks to the AC cross-field correlation $\widetilde{f}(x, \omega ) = \widetilde{E}_{Ref}^*(x, \omega ) \, \widetilde{E}_{in}(x, \omega )$. The input spectrum can be analytically derived and its spectral phase can be estimated through an iterative algorithm. The reference spectrum can similarly be derived analytically from the measured signal, as well as determined from the input pulse. The spectral width of these different spectra allows to calculate a robust quality criterion of the measurement from the measurement itself. This in situ verification capability is required especially for low repetition rate or single-shot measurements. The temporal resolution of the measurement is intrinsically matched to the pulse, as long as the entire spectrum, with a 1/e² bandwidth of $\Delta \omega$, of the pulse is measured, by $\delta t = \frac{2}{\Delta \omega }$. The spectrum of the reference pulse is wider than the original pulse and can sometimes exceed the spectral acceptance covered by the spectrometer. This is not a problem for the pulse itself but must be considered for the validity check of the measurement by including the spectral cut-off in the evaluation. The principle temporal range, defined by $\Delta T = \frac{2}{\delta \omega }$, with $\delta \omega$ being the spectral resolution of the spectrometer, of the SRSI method itself was already well suited for a measurement of the pulse beyond the temporal range of typical linear intensity scaling methods. The 2D extension enlarges the temporal range even further by eliminating the limitation of temporal aliasing between AC and DC terms, which in the 1D case limits the temporal window by a factor of three. Thus, the temporal window in the 2D case is only determined by the resolution $\delta \omega$ of the spectrometer. More interestingly, the measurement dynamics in the time domain can be increased compared to the detector dynamics thanks to two specificities of this measurement method. The first is that the measurement fully exploits the dynamics of the detector, if the spatial-spectral interferogram covers the entire detector. Then the measurement is converted into the domains of interest, which are the far field and temporal domain, by Fourier transformation. As the pulse is compressed close to its Fourier limit in these two domains, the measurement dynamics benefits from a double gain compared to the detector dynamics, proportional to the number of pixels in each dimension. Thus, a 16 M pixels camera, having >10$^7$ pixels, allows a gain of more than 10$^6$, depending on the filling of the intensity depth per pixel. The second feature of this method is that the AC term represents a cross-correlation of fields, and not of intensity. This particularity allows to gain an additional factor of two on the logarithmic scale of the intensity. In addition, it allows to study other properties, namely the distinction between coherent and incoherent contributions and spatial-spectral-temporal laser pulse distributions (e.g. spectrograms). On the other hand, the full dynamic exploitation over the whole temporal window can be limited due to the point spread function. With state-of-the art cameras ranging in the tens of megapixels resolution and pixel sizes well below 5 µm those are now dominantly defining the point spread function of the system. This requires a more careful and complex data treatment.

## 2D SRSI practical realization and calibration

**Fig. 2:**
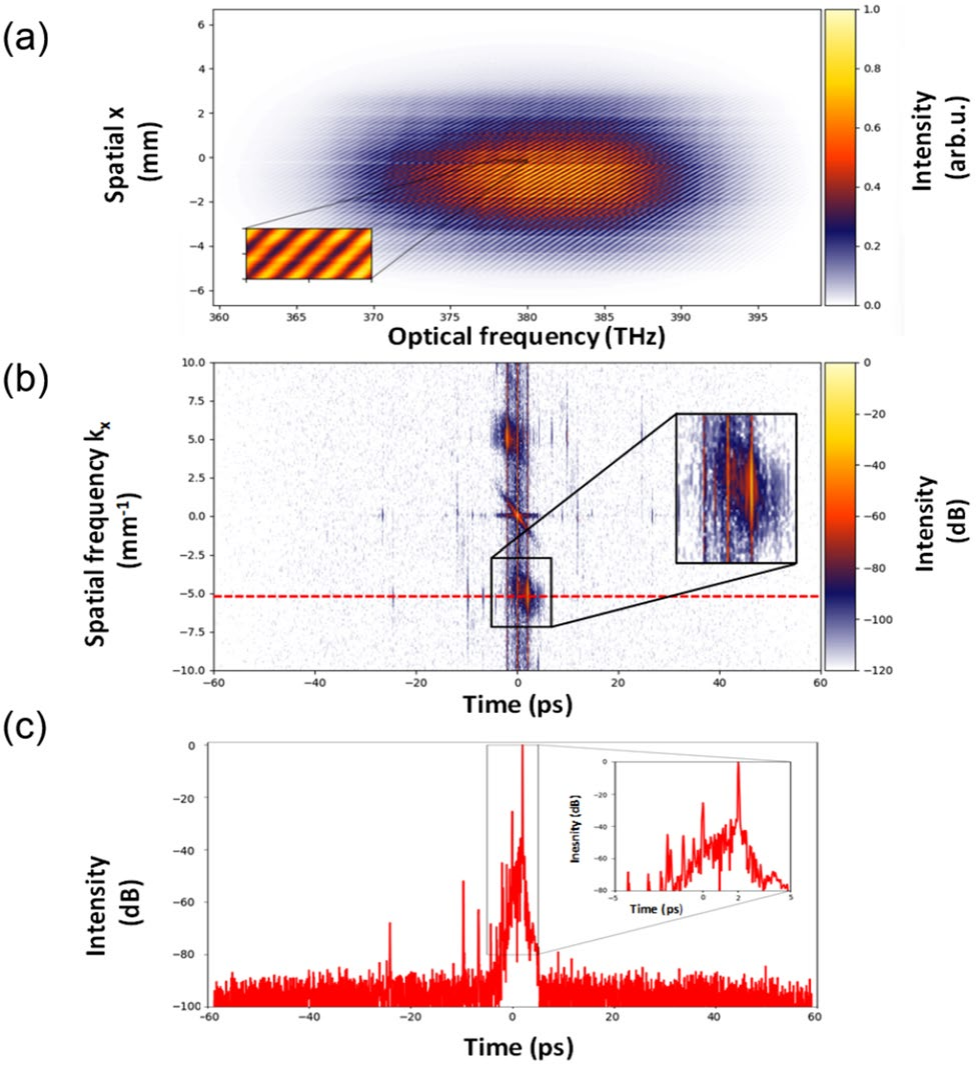
Data acquisition and treatment of 2D spatial-spectral interferograms. (**a**) Experimental spatial-spectral interferogram, (**b**) far-field- temporal intensity in log scale (dB) of the 2D Fourier transform of (**a**), insets shows the AC term as zoom in. Clearly the dominant pre-pulse is the artefact from the DC term. (**c**) temporal intensity profile of the AC term along the red dotted line through the maximum peak in (**b**) in log scale, inset shows the central part of the temporal window over +/−5 ps. Again, the dominant pre-pulse at zero is the artifact from the DC term.

To fully exploit novel capabilities, particular attention must be paid to the quality of the 2D SRSI experimental setup, which relies on a dispersion balanced Mach-Zehnder interferometer (see fig.1). While the first arm generates the optical SRSI signal, the second is used for delay and reference generation. This reference arm includes an imaging telescope, built from spherical mirrors with a thin ($\sim$1 mm) CaF2 crystal in its center for efficient XPW generation between crossed polarizers. Optimal signal-to-noise ratio in single-shot operation requires pulse energies of about 100 nJ in the reference beam obtainable with about 100 µJ at the interferometer input. The two beams, signal and reference, are directly recombined on the spectrometer slit with a small relative angle in the plane of the slit axis. This set-up is versatile, and depending on the choice of the final (focusing) mirror, the complete beam or only line-outs can be analyzed. Compared to the previous set-up^26^, the one presented here benefits from improvements on the quality of the optics (reflectivity, coating type, damage threshold), from imaging of the signal arm and a spectral calibration routine of the spectrometer using a higher number of reference lines and a more precise fitting of pixels to wavelength on the whole detector, from an extended delay line and especially from a spectrometer with a wide field of view (Princeton Instrument IsoPlane-320 A with extended image field) allowing to take full advantage of a large area camera detector like the MN34230 CMOS chip. Its detection matrix features a low read-out noise (1.2 electrons), a large depth of pixel (20000 electrons) and a high number of pixels (4656 x 3520 resulting in 16.4 Megapixels) with a size of 3.8 µm. The novel setup combines a significantly increased temporal excursion of 120 ps due to the improved spectral resolution of 0.02 nm/pixel at 800 nm, and an increased dynamic range of measurement of one to two orders of magnitude due to the more advanced camera detector. This setup, especially applying the higher resolution detector, was tested with the pulses of the CPA1 stage of the DRACO high-power laser system^35^. A detailed description of the system, including the contributions to coherent and incoherent contrast can be found in the “Methods” section. The spatial-spectral image of the beam (fig.2.a) is entirely included in the detector area allowing a measurement over this entire spatial dimension at the expense of contrast dynamics. In case of a full coverage of the detector with equally high signal close to the pixel dynamic limit, e.g. by only using the central part of the beam, the dynamics for contrast measurement could be maximized. Applying 2D Fourier transformation, we obtain fig.2.b in logarithmic scale (dB) displaying the central part in the spatial frequency domain showing the separation of the DC and the two AC terms as a consequence of the tilted beam arrangement. We observe details in these spatial-temporal domains to which we will return later. The vast majority of the Fourier transformation image is empty. All the signal information (or energy) is concentrated on the main DC and AC terms. The temporal profile (the square of the absolute value of the AC term) along the line passing through the highest AC peak marked in dotted red in fig.2.b is depicted in fig.2.c. This temporal intensity profile demonstrates a dynamic range of over 80 dB in a single acquisition and a 120 ps time excursion. The most dominant pre-pulse like signature on this curve is the one at zero of the temporal window. It is a measurement artifact resulting from the DC term in the k-t-space or Fourier domain. Similarly, a second measurement artifact symmetrical to the main pulse appears at a delay of −3 ps. Looking at the image in fig.2.b, it appears that the spatial profiles are not as clean as the temporal profiles. The main peaks of the DC and AC terms extend vertically over most of the k-t-space and thus overlap in the spatial frequency, or far field, domain. As there are two symmetric AC terms, any of them can be chosen equally for further analysis. In the following we will choose the lower one. Symmetry also means that the direction of the time is not obvious, in other words it is not intrinsically clear which side represents pre- and which post-pulses. Introducing a known additional delay between signal and reference arm allows both to determine the time direction and to check the temporal calibration. Equally a known pre- or post-pulse could be applied artificially, e.g. by a double reflection on a thin glass plate.

**Fig. 3:**
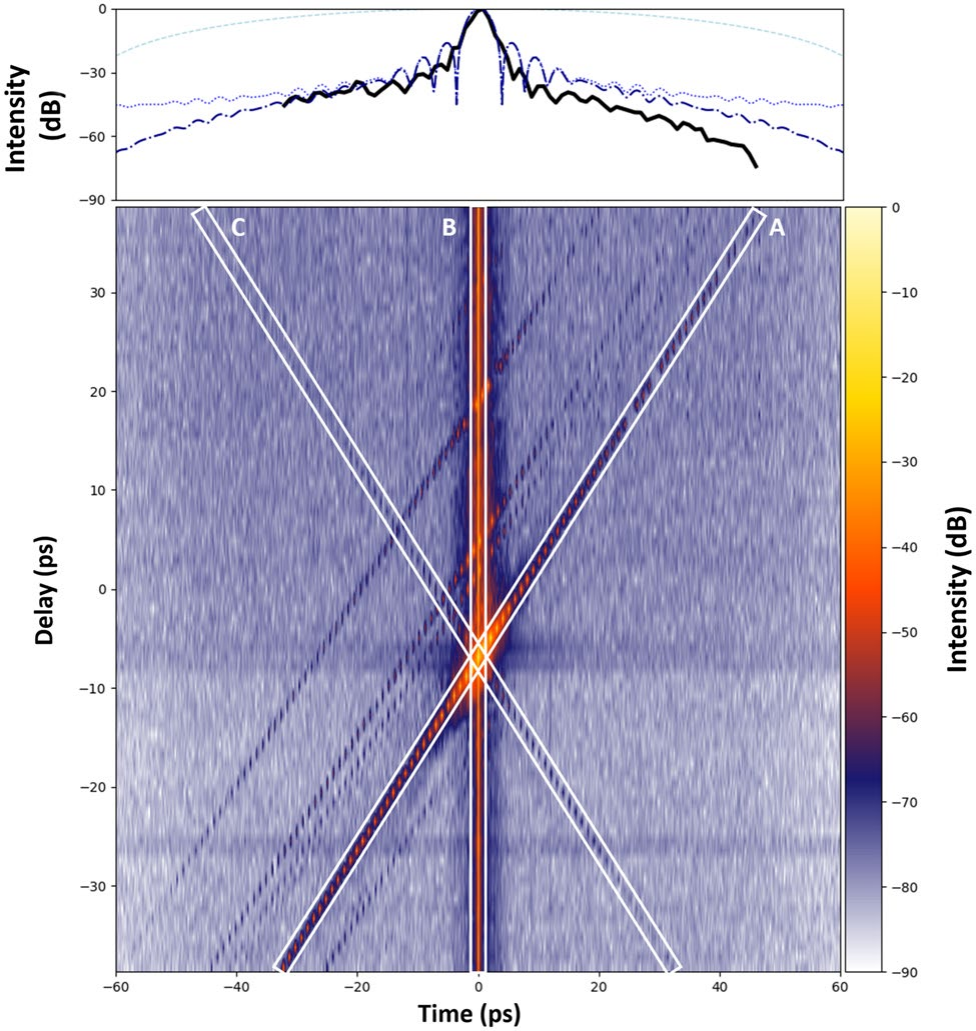
Experimental image of temporal intensities in log scale for a delay scan. Each line is the temporal intensity obtained for a given delay. The top graph shows the temporal envelopes, the black thick line is the measured envelope by the data along the line A, the light blue dash line is the pixel size contribution simulation, the blue dotted line is the slit size contribution simulation and the dark blue dash dotted line is the combination of pixel and slit contributions simulation. Further details in the text.

To validate the temporal range, the delay line was tuned and a series of measurements spaced with a delay of 1 ps performed. The interferogram of each measurement (like the one in fig.2.a) is processed to obtain the temporal profile in the far field (like in fig.2.c), displayed as individual lines in fig.3. We observe the progressive shift of the main pulse along the line A according to the applied delays. The other signal components (post pulses, pre-pulses and pedestal) follow in the same way. The changes on the delay line allow to check both the temporal calibration and directionality. The strong signal along the vertical line B represents the leakage (or spread along the spatial frequency axis) of the DC component. Finally, the leakage of the symmetrical AC component appears along the line C. As the positions of these two artifacts are known, they can be easily eliminated when evaluating the temporal contrast behavior. The profile along the line A corresponds to the temporal envelope related to the point spread function (PSF) of the spectrometer. The accuracy of this evaluation suffers from the delicate alignment (angle between the two arms) in the spectrometer slit which seems to have slightly varied in this measurement. In spite of this measurement bias, mainly expressed by the asymmetry of the curve around its maximum, we note that the envelope is maximum around time zero of the temporal window. It decreases rather quickly to a level of about −40 dB, followed by a much slower decrease over time. Thus, no temporal aliasing is observed, which would be indicated by a strongly chirped pre- or post-pulse appearing when the main pulse is leaving the temporal window, due to the attenuation of the delayed main pulse, or alias, below the noise floor. This behavior differs from the one of the previous device^26^ where the larger pixel size corresponded to the resolution of the spectrometer (typ. 26 µm). The new detector has seven times more resolution, or seven times smaller pixels (typ. 3.8 µm). Consequently, the temporal envelope is no longer limited by the detector but by the response function of the spectrometer. This response has, as shown by this measurement, components that are certainly much weaker (−40 dB) but of higher frequency than expected in the previous setup. The main consequence of this envelope is that the measurement dynamics varies with the delay. It is maximum near time zero of the temporal window, but it is precisely there that the DC term introduces the most disturbance. In consequence the measurement has its maximum dynamic close to zero of the temporal window but outside the area disturbed by the outreaches of the DC term. Therefore measuring the main pulse requires a slight shift of it relative to the zero, or center, of the temporal window. Depending if the interest lies on the pre- or post-pulse side, the DC outreaches can be placed on the other side respectively. Another consequence is the possibility to shift the measurement window arbitrarily with the optical delay line without experiencing aliasing of the main pulse due to its attenuation by the PSF, while knowing that the highest dynamic is always reached only in the temporal center of the individual shifted time window. The study of pre-pulses beyond 120 ps of the temporal window is therefore in principle possible.

**Fig. 4:**
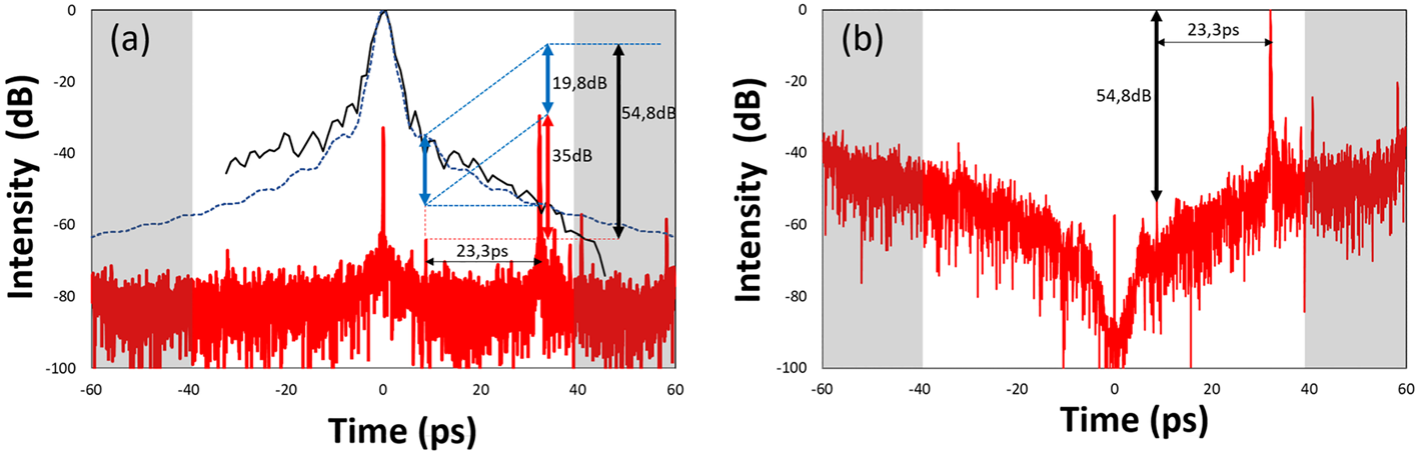
Impact of the PSF. (**a**) temporal intensity in log scale in red, temporal envelope due to PSF of the spectrometer in blue dotted line for the simulation, the black one is the experimental measurement. (**b**) Temporal intensity profile with temporal envelope correction by simulation curve of (**a**). The greyed areas are marking the in practice not usable temporal areas due to the reduced dynamic range.

Correcting the temporal envelope to correct the temporal intensity measurement requires precise determination of the envelope over the entire measurement dynamic range and excursion. To understand the temporal envelope origins and main relevant parameters, a theoretical model is used to simulate it. The temporal envelope depends upon the point spread function of the spectrometer onto the detector and its pixel size and pitch through Fourier transform^32,34^. These influences of the spectrometer’s signal can be expressed by using a convolution between rectangular functions for the pixels and for the spectrometer’s slit, the point spread function of the spectrometer and the input signal

$$\widetilde{M}(x, x_\omega ) = \sum _{i=0}^{N} \sum _{j=0}^{M} \left( \widetilde{PSF}(x, x_\omega ) \, \bigotimes \text {Rect} \left( \frac{x_\omega - j x_{j\omega _{slit}}}{x_{j\omega _{slit}}} \right) \, \bigotimes \text {Rect} \left( \frac{x - i x_{pix}}{\delta x_{pix}} \right) \, \bigotimes \text {Rect} \left( \frac{x_\omega - j x_{j\omega _{pix}}}{\delta x_{j\omega _{pix}}} \right) \, \bigotimes \widetilde{S}(x, x_\omega ) \right) ,$$

where $\widetilde{S}(x, x_\omega )$ is the input signal due to the input beam, $$\text {Rect}(x) = {\left\{ \begin{array}{ll} 0, & \text {if } |x|> \frac{1}{2} \\ 1, & \text {if } |x| \le \frac{1}{2} \end{array}\right. }$$, where $x_{j\omega _{pix}}$ and $x_{pix}$ are respectively the pixel size equivalent in the spectral and spatial domains, $x_{j\omega _{slit}}$ is the spectrometer’s slit width in the detector plane, $\widetilde{PSF}(x, x_\omega )$ is the point spread function of the spectrometer, which is the response obtained for a Dirac delta function input. Note that $\delta \le 1$ expresses the filling ratio of the pixels. The spectral dimension $x_{j\omega }$ is used instead of $\omega$ directly to take into account the non-linear dependence over $\omega$ of the $\widetilde{PSF}$ and pixel size and pitch. Indeed, as the spectrometer uses a grating, its dispersion follows the grating rule that is proportional to the wavelength and not the frequency. Thus, the dependency of the pixel size and pitch follows a nearly constant rule in wavelength meaning a quadratic dependence in optical frequency $\omega$. It is one of the reasons of complexity of the temporal envelope shape that is not a single sinc function but rather a sum of different sinc functions with different widths. The same effect applies to the perfect image of the spectrometer’s entrance slit on the detector. Finally, the most complex contribution comes from the PSF, which varies with both *x* an $x_\omega$. Then, in the temporal domain, after selecting the AC component in the spatial frequency domain, the pixelization and the PSF effects can be translated onto the temporal envelope as

$$M\left( t_{x_\omega }\right) \sim S\left( t_{x_\omega }\right) \sum _{j=0}^{M}\left( PSF\left( t_{x_\omega }\right) \cdot \text {sinc}\left( {x_{j\omega }}_{\text {slit}} \cdot t_{x_\omega }\right) \cdot e^{2i\pi j{x_{j\omega }}_{\text {slit}} \cdot t_{x_\omega }} \cdot \text {sinc}\left( \delta {x_{j\omega }}_{\text {pix}} \cdot t_{x_\omega }\right) \cdot e^{2i\pi j{x_{j\omega }}_{\text {pix}} t_{x_{\text {pix}}}} \right)$$

where sinc(*x*) is the sinc function sin(*x*)/*x*, and $t_{x_\omega }$ is equivalent to time. This formula is an approximation, since the time dimension is distorted by irregular sampling in $\omega$. This irregular sampling in frequency also scrambles the temporal sinc envelope by removing the regular zeros of the sinc shape at the black curve on the positive delay side in the upper plot of fig.3. This envelope modification is spectrum dependent. To measure the temporal envelope, since the dynamic range should be higher than 10$^5$, the calibration cannot be carried out using the usual narrow-line calibration sources. As the calibration is spectrally dependent it has to be done with a source with the same spectrum as the pulse to be measured. Thus, the main pulse itself can be used in the 2D SRSI configuration by sweeping the delay line and recording the main pulse peak level as the optimal practical method. Attention has to be paid that the temporal sweeping using the delay line is not producing pointing variations. This pointing variation is the cause of the asymmetric shape on the experimental data (black thick line) in fig.3, while theoretically the envelope is symmetric. The light blue dotted line is the envelope due to the pixel size corresponding to the full temporal excursion of 120 ps. The blue dashed one is the contribution of the slit. The combination of both (dark blue dash dotted line) is already close to the experimental curve. The differences between theoretical estimation and measured calibration also highlight that the contribution of the PSF on the noise level distribution is more complex and smoother than a pure sinc shape. The main cause of this smoothing is a higher variation of the PSF along the detector. To verify the dynamic range of the measurement, a Mach-Zehnder interferometer is used to insert a pre-pulse of known attenuation, The pre-pulse is generated just before compression and thus no significant nonlinear effects are expected. The measurement shown in fig.4.a is performed with an attenuation of 57 dB (two calibrated optical densities of 28.5 dB each at 800 nm). The Mach-Zehnder interferometer is set in a way that the generated pre-pulse precedes the main pulse by 23.3 ps, which is sufficient to avoid the typical pedestal around the main pulse. The 2D SRSI delay is adjusted to position the pre-pulse close to the zero position of the temporal window, but avoiding the contributions of the DC term, as described above. In fig.4.a. the pre-pulse is observed 23.3 ps before the main pulse as expected, but the ratio between the measured attenuation reads 35 dB instead of 57 dB corresponding to the applied attenuation. This deviation is related to the temporal envelope due to the PSF drawn as the blue dotted line for the simulation and the black solid line for the measurement discussed with fig.3. By correcting the contribution related to the temporal envelope the expected 57 dB ratio of pre to main pulse can be recovered to 54.8 dB. The PSF corrected curve (fig.4.b) then allows for the direct display of the relation. Likewise, it is figuratively shown here that the noise level is depending on the temporal position relative to the zero position of the temporal window. The noise floor is at its lowest where the envelope is maximum, i.e. around the center of the temporal window. Measurements after 40 ps and before −40 ps suffer from reduced dynamic range represented by the grey areas in fig.4. Calibrating the temporal envelope remains a delicate task, in particular as the spectrometer features an adjustable slit used to optimize the signal level and the slit width is one of the most important parameters influencing the temporal envelope.

**Fig. 5:**
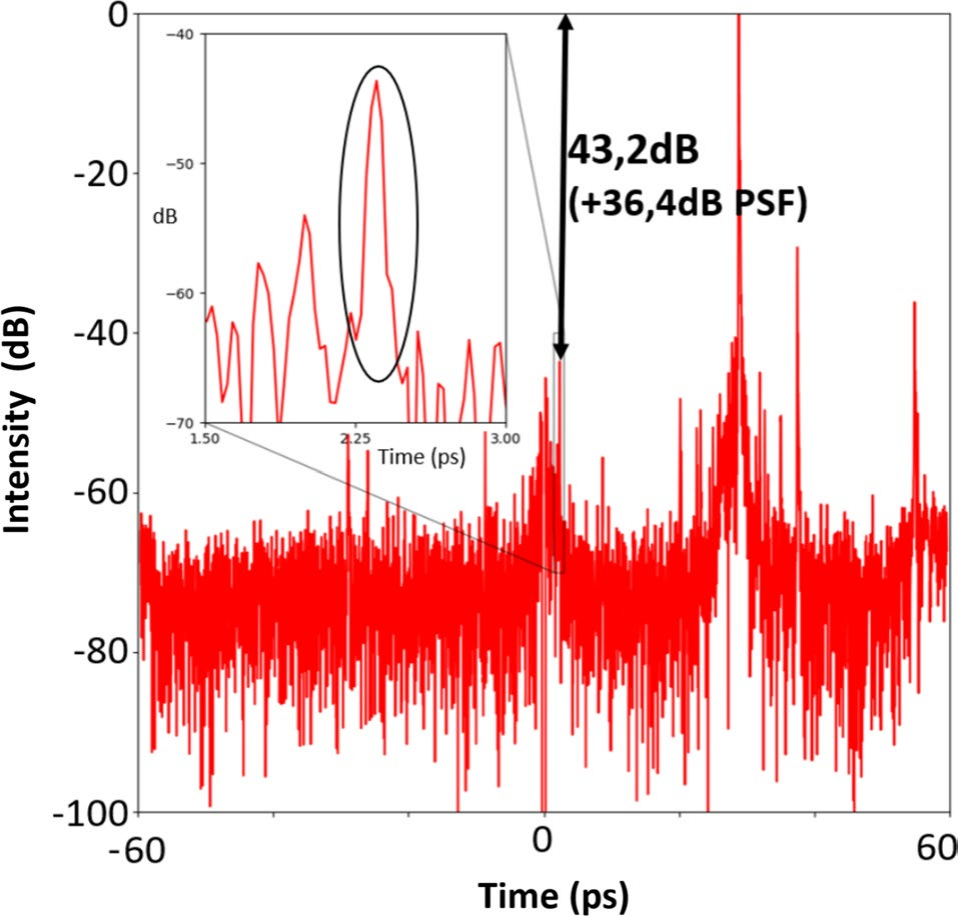
Experimental demonstration of a dynamic range. Shown is a 79.6 dB measurement for a predefined test pulse attenuation of 80.5 dB.

Thus, the maximum dynamic range is expected when the pre- or post-pulse features to be analyzed are close to the central zero position. Experimentally the best dynamic range was obtained by measuring a pre-pulse set to 2.25 ps in the temporal window, 22.5 ps before the main pulse and attenuated by an optical density of 8.05 (−80.5 dB) at 800 nm (fig.5). This pre-pulse is still clearly visible above the noise level in single shot mode indicating a maximum possible dynamic close to or exceeding 90 dB.

## Measurement of spectral-temporal features

**Fig. 6:**
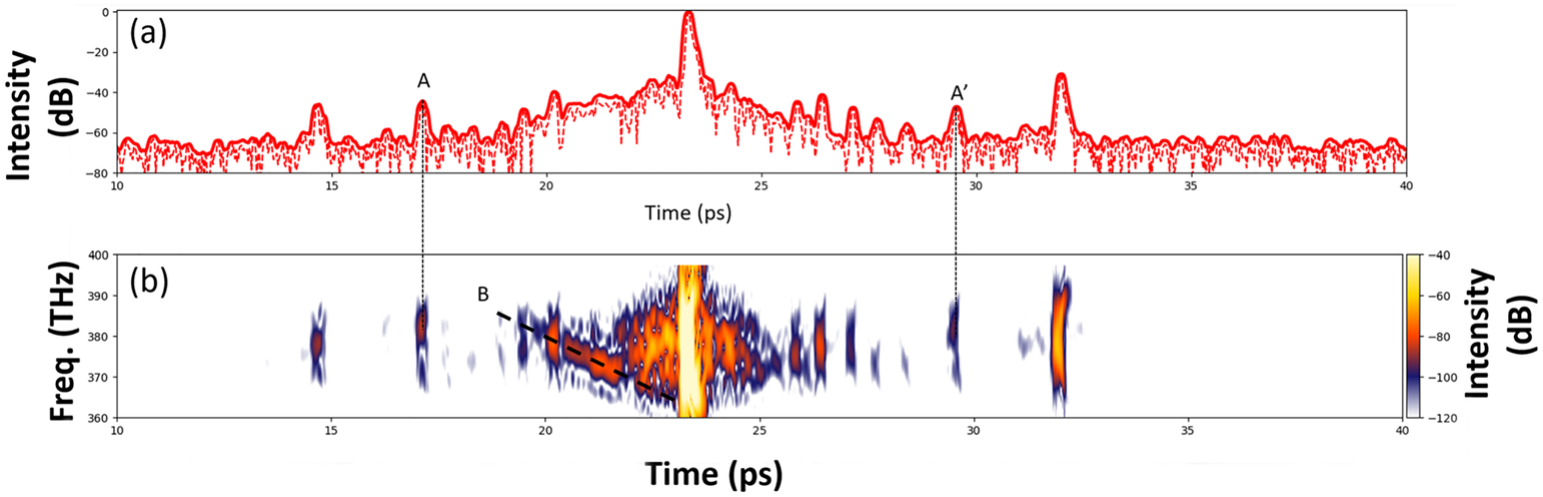
Spectrogram. (**a**) Temporal intensity in log scale (red dashed line), and gated temporal intensity by using a gate of 200fs (thick solid red line). (**b**) Corresponding spectrogram of the pulse in log scale as described in the text.

Another feature of the 2D SRSI technique is the access to the temporal distribution of the complex field (i.e., its amplitude and phase) by step-wise filtering, or gating, in the temporal domain and back-transformation to the spectral domain for the specific temporal steps. Thus, spectrograms, representing the spectral evolution over time, of any pulse feature like individual pre- or post-pulses can be retrieved for single shots with a dynamic range of better than 80 dB. This capability can be used to detect and analyze phase effects or wavelength shifts, e.g. for pre-pulse A on fig.6.a. Although on the temporal profile this pre-pulse looks identical to others, on the spectrogram derived from the same data (fig.6.b.), a blue shift can clearly be identified. The same blue shift is also seen on the symmetric post-pulse A’. In the pedestal of the main pulse we observe contributions that seem to interfere in time and space with each other. In particular the one marked with B shows a significant negative chirp. This unprecedented measurement capability is essential to identify the origin of these contributions and thus to subsequently improve the temporal contrast of high-power lasers.

## Coherent dynamic enhancement

**Fig. 7:**
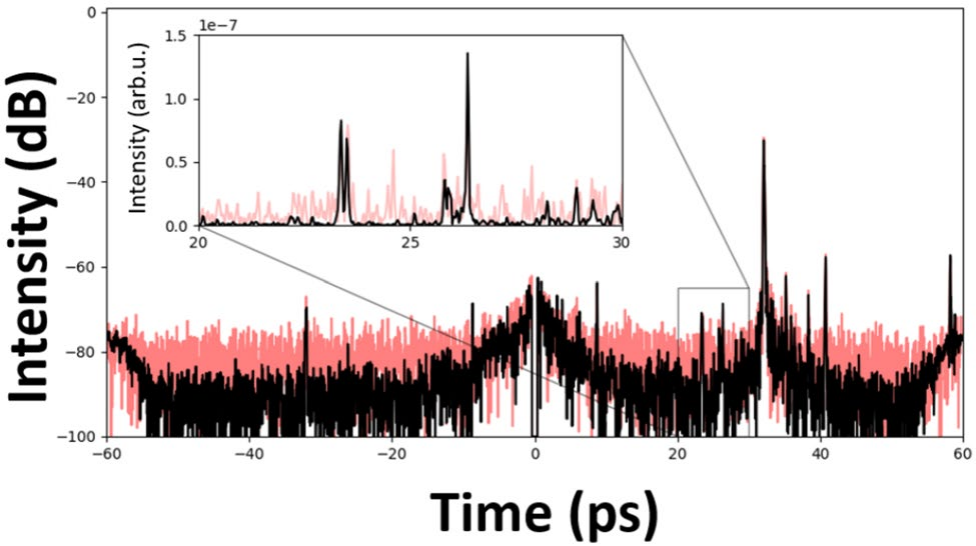
Impact of accumulation of shots. Temporal intensity in log scale in red for a single measurement and in black for an accumulation of 10 measurements. The inset zoom is in linear scale for the intensity. Note that the DC component has been filtered out.

All previous analysis was made with data from a single pulse. Although very high-power lasers typically operate at a low repetition rate, it is possible to make successive measurements or to integrate several successive pulses. The first will be called accumulation of measurements, the second accumulation of pulses. The accumulation of pulses allows to increase the signal on the detector. As it preserves the measurement dynamics of the detector, no increase in the signal with respect to the measurement noise of the detector is gained. But the coherent parts of the signal are increased linearly in comparison to the incoherent contributions, for example the pre- and post-pulses with respect to the amplified spontaneous emission (ASE). In other words, the ASE contributions will be linearly damped in the measurement with the number of accumulations of pulses, while the coherent pulse contributions are maintained. The accumulation of measurements retains this characteristic of increasing the dynamics of the coherent contributions compared to the incoherent, but it also allows to additionally gain on the dynamics of the detector. The noise of the detector in this case is equivalent to any incoherent contribution. The overall signal-to-noise ratio thus increases linearly with the number of measurements accumulated. Unlike other techniques used for contrast measurement, the 2D SRSI method is a cross-correlation of field and not of intensity. This distinction is important for the dynamics of the measurement. It explains the increase of the linear dynamics with the number of measurement accumulations, where most other methods see their dynamic increase with the square root of the number of acquisitions. The experimental verification with ten accumulations is shown in fig. 7. The lower line exhibits a 10 dB increase in dynamics for this mode. The comparison is made with a single measurement. A zoom window on pre-pulses shown in a linear scale highlights the decrease of the noise level and that some pre-pulses below the noise level can now be resolved. It is thus possible to increase the dynamic resolution of the measurement by 10 or even 20 dB (with 100 measurements). Measurements made by TOAC often contain 1000 or more measurements. Under such conditions the 2D SRSI method allowed to obtain a gain of 30 dB and thus a dynamic range equivalent to 110 dB.

The experimental measurements shown in fig.7 confirm that the coherent contribution is not modified by the accumulation, while the incoherent contributions are proportionally decreased. Thus, this method offers a possibility to measure pre- or post-pulses that are even below the ASE and/or shot noise level by accumulating successive measurements, provided their origin is persistent.

## Conclusion

The 2D SRSI (SRSI-ETE) method offers a unique combination of single-shot pulse characterization properties over the entire spectral range of interest, i.e. with a problem adapted temporal resolution, amplitude and phase for the main pulse, temporal contrast in the far field over an excursion of 120 ps with a demonstrated singe-pulse dynamic range exceeding 80 dB. The implementation of the presented detector with increased resolution has demonstrated the possibility of extending the time excursion by almost a factor of eight and the dynamic range in the order of 10 dB. This raised the question of the spectrometer’s PSF correction to fully exploit the dynamic range of this measurement and triggered the design of an adapted spectrometer for future use, not discussed here. The revised device demonstrates the absence of temporal aliasing, an increased dynamic range, the ability with this dynamic range to observe spectrograms on pre- or post-pulses and the ability to distinguish incoherent and coherent components thanks to the accumulation of measurements. Self-referenced interferometric ultrafast pulse measurement techniques that provide access to the full electric field (amplitude and phase) so far typically addressed two orders of magnitude dynamic range, presented on a linear scale. In 2009, Witting et. al^36^. introduced the “SEA-CAR-SPIDER” scheme and presented a reconstruction of a 54 fs pulse, derived from spectral amplitude and phase, displayed on a logarithmic scale with a dynamic range of approximately three orders of magnitude. To further advance this field, future studies could benefit from a systematic comparison of 2D SRSI with other two-dimensional approaches (e.g. SEA-F-SPIDER, SEA-CAR-SPIDER^36,37^), in particular in light of the here observed strong influence of the spectrometer’s point spread function (PSF). To date, the 2D SRSI method remains unique in its combination of the auto-heterodyne measurement principle and the spatial-spectral interferogram. This allows for a comprehensive characterization of the electric field across a broad temporal window, a wide dynamic range, one spatial dimension and the full spectral bandwidth of the laser, in both single-shot and multi-shot configurations (see Table 1 in the “Methods” section for a comparison of state-of-the-art techniques). The 2D SRSI technique enables not only comprehensive characterization of the temporal intensity contrast at focus, incorporating all spatial and spectral degrees of freedom, as well as advanced multidimensional characterizations, such as spectral-temporal (spectrogram), spatial-temporal, and spatial-spectral measurements. The capabilities of 2D SRSI make this method an essential tool in particular to continue the quest for more powerful and temporally cleaner pulses of modern high intensity ultra-short pulse laser systems.

## Methods

### Description of the DRACO laser system and elements influencing pulse temporal contrast

**Fig. 8:**
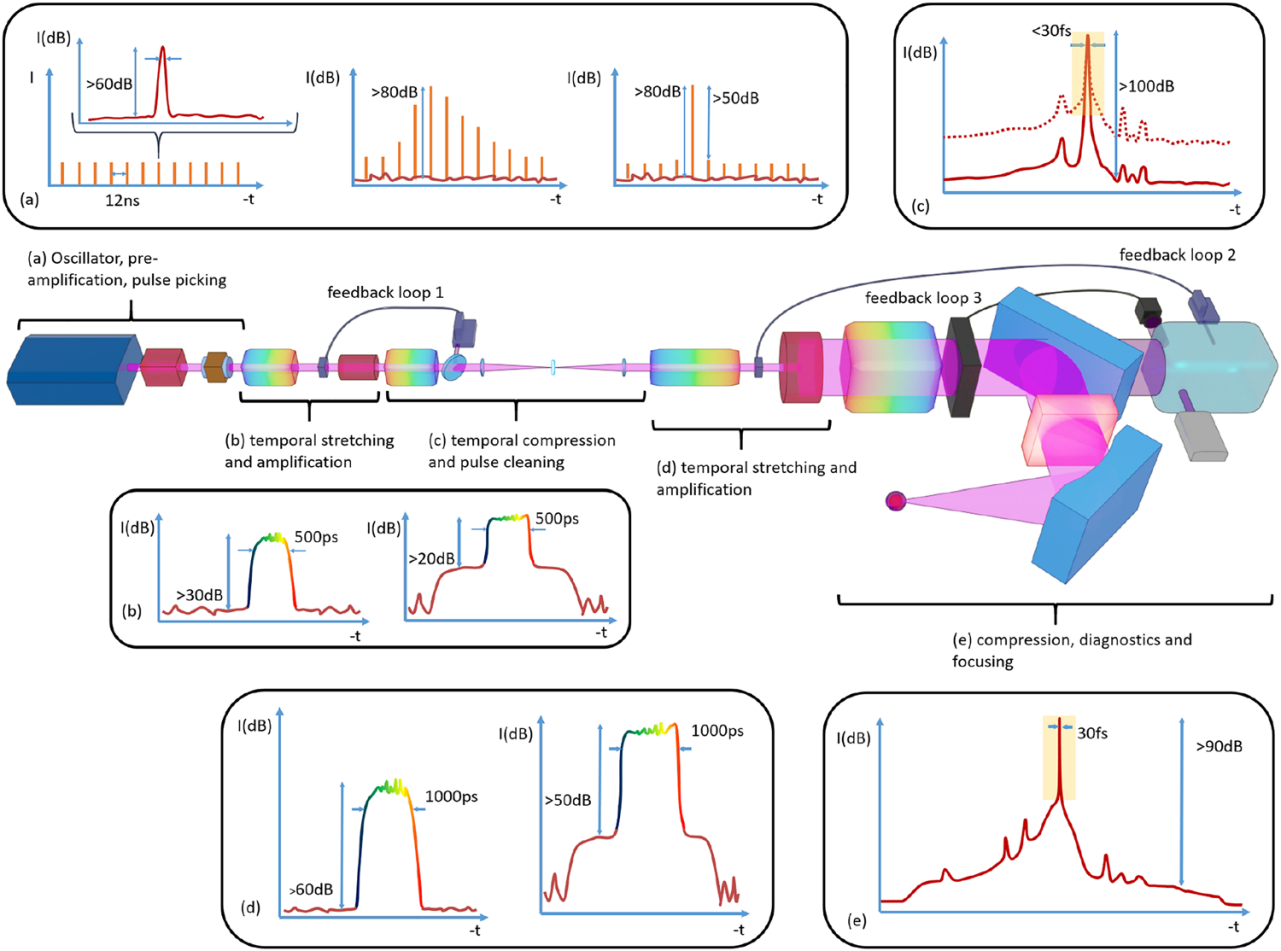
Illustration of the temporal intensity pulse evolution of a double CPA laser chain. Detailed explanations can be found in the text and quantitative sample data represents the DRACO system at HZDR. (**a**) The optical oscillator is followed by a booster pre-amplifier and a pulse selection device reducing the repetition rate from the MHz scale to the 10 Hz scale. (**b**) The pulses are temporally stretched and amplified to mJ energy level. (**c**) An intermediate compression and temporal contrast cleaning unit separates the two CPA stages. (**d**) In CPA2 contrast improved laser pulses are carefully stretched and amplified to the 10 s of joule level. (**e**) Final compression leads to PW class laser pulses. These pulses undergo pulse characterisation (ideally on-shot) and are focused for their application.

As source for the presented experiments the CPA1 probe beam of the high-intensity laser system Draco has been used. The Draco system is a classical double-CPA system. The architecture of such CPA lasers is complex and continuously evolving. The objective of these sources is to provide the highest possible focal intensities above 10$^{21}$ W/cm², exploiting ultra-short laser pulses of 20–30 fs duration focused to diffraction limited spot size^14^. The level of tolerable temporal pulse contrast depends on the experiment, and is for laser-solid interactions typically limited by the onset of target ionization (i.e. the dielectric breakdown intensity for dielectrics^10,11^. Current solid target applications requiring the highest intensities are proton or ion acceleration^7,9,12,13^. To avoid uncontrolled pre-plasma generation and expansion, the intensity before the main pulse should not exceed 10$^{12}$ W/cm² to 10$^{14}$ W/cm² corresponding to characteristic time intervals of 10 ps to 30 fs^10^. Thus, temporal contrast requirements grow with peak intensity. For a 1 PW system focused with short focal length optics, the corresponding intensities approach 10$^{22}$ W/cm², resulting in a contrast requirement exceeding 10$^{10}$ (100 dB) on the few 10 ps scale. Fig. 8 depicts a typical contrast optimized double-CPA laser system, using the principle example of the Draco laser system. Ultrashort pulses are generated by a mode-locked oscillator yielding low pulse energy ($\sim$10 nJ), ultra-short pulse duration ($\sim$10 fs) and high repetition rate ($\sim$78 MHz). The peak power is still low ($\sim$MW). Before selecting a pulse from the pulse train for chirped pulse amplification^38^, the pulse is pre-amplified by a booster amplifier. This amplification is done directly on the pulse train and raises the energy level of the pulse, afterwards selected and filtered by fast Pockels cell combinations, to the microjoule level range (Fig.8(a)). This pre-amplification allows to limit the amplification needed in the first CPA stage and thus the amplified spontaneous emission (ASE) level in the stretched pulse. The selected pulse is stretched and shaped in the temporal domain. The chirped pulse is amplified from the 100 nJ level to the few mJ level (fig.8(b)) and re-compressed. A portion of the beam is used to measure and control the temporal shape by an active feedback loop ensuring compression to its Fourier transform limit (feedback loop 1 in fig. 8), using self-referenced spectral interferometry (SRSI), or similar spectral phase retrieval techniques, for measurement and acousto-optic programmable dispersive filters (AOPDF) for pulse shaping. The optimized pulse, despite its perfect compression, still carries distortions due to coherent contributions, such as high frequency spectral amplitude or phase ripples, pre- or post-pulses, or incoherent contributions, such as the ASE pedestal. Transmission optical elements can generate post pulses in the 10 s of picosecond range, which can couple to pre-pulses due to non-linear effects in the materials of the laser chain^35,39–41^. These contributions can only be seen using high dynamic range temporal contrast diagnostics whose temporal measurement range is larger than the pulse measurement device used for the feedback loop (represented as a rectangle on the temporal intensity in fig.8(c)). To suppress these temporal contrast deteriorations of the first CPA stage, providing mJ class laser pulses, the dual CPA architecture utilizes a nonlinear cross-polarized wave (XPW) generation filter^42^. This temporal cleaning method allows to gain orders of magnitude on the temporal contrast as indicated by the temporal intensity represented in logarithmic scale (fig.8(c)). The transmission of the nonlinear filter is of the order of 10 $\%$, which means that the energy of the cleaned pulse can be between 100 µJ and 1 mJ. For stable operation, the repetition rate of the first CPA chain is chosen between 10 Hz and 1kHz, which results in an average power of 10 mW to 1 W. This repetition rate facilitates the use of scanning temporal contrast diagnostics like third-order auto-correlators (TOAC)^15^. This temporally and spatially cleaned pulse is used to seed the second CPA chain. The pulse is shaped and stretched in time before amplification to an energy level of up to tens of Joules at a low repetition rate (<1 Hz). Care has to be taken to the surface quality of stretcher optics, else compromising ps contrast^24,43–46^. To avoid optical nonlinearities in the amplifiers, the beam is temporally stretched to typically 1ns (fig.8(d)). This large stretching rate, while maintaining the need for a compressed pulse shape close to the Fourier transform at the end, implies a second feedback loop on the temporal shape (feedback loop 2 in fig.8). Even with these large stretching factors, the pulse needs to be spatially expanded step-wise in consecutive amplification stages to tens of centimeters to avoid laser induced damage. Often, such multi-pass power amplifier stages are separated by active Pockels cells, further suppressing ns pre-pulses potentially generated in these amplifiers and preventing damage from back-propagating pulses. Such wide beams, in order to keep the final focus close to the diffraction limit, require at least one deformable mirror-based feedback loop (loop 3 in fig.8) before and/or after pulse compression. Exiting the large aperture compressor, the beam is transported to the experimental area where it is focused onto the target. A re-collimating plasma mirror device can be inserted into the transport line improving the contrast for critical applications^47^.

### Comparison between the presented 2D self-referenced spectral interferometry and recently developed or commercially available reference instruments

See Table 1.

**Table 1:** Comparison between the presented 2D self-referenced spectral interferometry, its previous version (SRSI-ETE), recently developed high dynamic range single-shot cross-correlators (SSCC) and commercially available reference instruments (Sequoia, Tundra, Wizzler, FROG, SPIDER).

Technique	SNR	DR	TR [ps]	TRes [fs]	pts	NrS	bandw	SpP	SpT	Operating principle
2D SRSI (this work)	>10$^9$	>10$^8$	120	18	4656	1	Full pulse	Yes	Yes	XPW 2D SRSI, single shot, spatio-temporal information, high dynamic spectrogram
SRSI-ETE^26^	>10$^8$	>10$^5$	18	18	900	1	Full pulse	Yes	Yes	XPW SRSI-ETE, single shot, spatio-temporal information
SSCC-OPR^48^	>10$^6$	>10$^4$*	200	6260	35	1	SHG/THG limitations	No	No	Optical pulse replicator, single shot, *estimated in multiple shot measurements
SSC-OFG^19,49^	>10$^9$		114	200	160	1	SHG/THG limitations	No	No	OPA based, optical fiber array, single shot
SSC-OFG -NOPA^50^	>10$^8$	>10$^2$	14	800	17.5	1	NOPA limitations	No	No	NOPA base, optical fiber array, single shot
SS-FOA^21^	>10$^{10}$	>10$^4$	65	160	406	1	SFM limitations	No	No	XPW+SFM
SEA-CAR- SPIDER^36^	>10$^3$	>10$^2$	4.8	<10	1280	1	Full pulse	Yes	Yes	SPIDER principle using spatially encoded interferogram, influence of PSF not specified
WIZZLER FASTLITE^28,29^	>10$^5$	>10$^4$	5	10	500	1	Full pulse	Yes	No	XPW SRSI single shot, spectral phase reconstruction
FROG SWAMP OPTICS	>10$^3$	>10$^2$	2	35	72	1	Full pulse	Yes	Part*	SHG single-shot, auto-correlation, spectrally resolved *FROG can have partial SpT.
SPIDER APE	>10$^3$	>10$^3$	1	15	>60	1	Full pulse	Yes	No*	SFM single shot spectral, shear interferometer *Custom SPIDERs can have SpT
SEQUOIA AMPLITUDE	>10$$^{10}$$	>10$$^8$$	>2000	>100	n.r.	2$$\cdot 10$$$$^3$$ (3$$\cdot 10$$$$^4$$)	SHG/THG limitations	No	No	Scanning third order CC
TUNDRA UFI	>10$^{12}$	>10$^8$	>2000	>100	n.r.	4$\cdot$10$^4$	SHG/THG limitations	No	No	Scanning third order CC

SNR: signal to noise ratio, DR: dynamic range (largest signal measured/smallest signal measured), TR: temporal range, TRes: temporal resolution, pts: number of sampling points, NrS: number of required shots per full measurement, bandw: measurement bandwidth, SpP: spectral phase measurement capabilities, SpT: spatio-temporal measurement capabilities, n.r.: not relevant.

## Data Availability

The datasets generated during and/or analysed during the current study are available from the corresponding author on reasonable request.
